# Association of the trimethylamine N-oxide with cardiovascular risk and vascular alterations in middle-aged patients with risk factors for cardiovascular diseases

**DOI:** 10.1042/BSR20232090

**Published:** 2024-05-24

**Authors:** Natalia Spasova, Desislava Somleva, Bozhidar Krastev, Radostina Ilieva, Angelina Borizanova, Dobrin Svinarov, Elena Kinova, Assen Goudev

**Affiliations:** 1 Department of Cardiology, University Hospital, UMHAT “Tsaritsa Yoanna - ISUL”, Sofia, Bulgaria; 2 University Hospital Alexandrovska, Faculty of Medicine, Medical University, Sofia

**Keywords:** Arterial Stiffness, Atherosclerosis risk factors, Carotid artery plaques, Intestinal Microbiota, Trimethylamine N-oxide

## Abstract

Background: Trimethylamine N-oxide (TMAO) is synthesized by the intestinal microbiota and is an independent predictor of cardiovascular disease (CVD). However, its underlying mechanisms remain unclear. We investigated TMAO levels across different CVD-risk patient groups, and evaluated associations between TMAO and vascular alterations (e.g., arterial stiffness, intima-media thickness [IMT], and the presence and grade of carotid artery plaques [CAPs]).

Methods: We examined 95 patients (58.5 ± 7.3 years): 40 with clinical atherosclerotic cardiovascular disease (ASCVD), 40 with atherosclerosis risk factors (RF), and 15 controls. Arterial stiffness was measured by Carotid-Femoral Pulse Wave Velocity (C-F PWV). B-mode ultrasound was used to evaluate the presence and grade of CAPs and carotid IMT (CIMT). TMAO was measured by high performance liquid chromatography-tandem mass spectrometry (LC-MS/MS) and results were presented as the median (interquartile range).

Results: TMAO levels were higher in patients with ASCVD (251.5 [164.5] µg/l) when compared with patients with RFs (194.0 [174] µg/l, *P*=0.04) and controls (122.0 (77) µg/l, *P*<0.001). A significant correlation was observed between TMAO and PWV (*r* = 0.31, *P*=0.003), which was not confirmed after adjustment for RFs. TMAO levels were significantly correlated with plaque score (*r* = 0.46, *P*<0.001) and plaque height (*r*=0.41, *P*=0.003), and were independent predictors for grade III plaques (odds ratio [OR] = 1.002, confidence interval (CI) 95%: 1.000047–1.003, *P*=0.044).

Conclusions: TMAO levels are increased with expanded CVD risk. Across different types of vascular damage, TMAO is associated with atherosclerotic changes.

## Introduction

Despite recent advances in predicting cardiovascular disease (CVD) risk and treatment benefits, CVDs remain the leading cause of morbidity and mortality worldwide [1]. There is now growing evidence that CVD development cannot be completely explained by traditional risk factors (RF) alone, and that a substantial proportion of patients often present with their first or recurrent cardiovascular event with a well-controlled RF profile, according to current guidelines [2]. Therefore, this emphasizes the need to examine and evaluate new factors and pathways contributing to CVD initiation and progression, with a view to providing new therapeutic targets to mitigate residual CVD risk.

Intriguing data exists on the interrelationship between a community of microorganisms inside our gastrointestinal tract (‘microbiota’) and CVD and health [3]. Intestinal microbiota, via varied metabolite production, is likely a key connecting factor between dietary habits and CVD risk. For example, trimethylamine N-oxide is a metabolite synthesized by the intestinal microbiota and has been established as an independent predictor of cardiovascular events [4]. However, its mechanism of action remains unclear, and TMAO causality in cardiovascular events remains controversial. Of the different types of vascular damage, current TMAO data posit connections to atherosclerosis processes, e.g., scavenger receptor expression and thrombocyte activation [5]. While this evidence has mainly come from experimental trials, a paucity of data exists between TMAO levels and the integrative assessment of vascular structure and function in clinical settings, which may provide insights on action pathways.

## Purpose

The purpose of our study was to investigate intestinal microbiota-derived TMAO levels across different CVD-risk patients and examine associations between TMAO and vascular alterations (arterial stiffness, intima-media thickness, and the presence and grade of carotid artery plaques [CAPs]) in these patients.

## Materials and methods

### Patient population and study design

In this cross-sectional study, we evaluated 95 consecutive patients, aged 40–75 years. Our cohort comprised patients with documented clinical atherosclerotic CVD (ASCVD), (*n*=40), patients with risk factors (RFs) for atherosclerosis (*n*=40) and controls (*n*=15).

### Inclusion and exclusion criteria

Inclusion and exclusion criteria are outlined (Table 1). Observers, who performed diagnostic instrumental and laboratory analyses were blinded to group assignments.

**Table 1:** Study inclusion and exclusion criteria

Inclusion criteria	Exclusion criteria
**Patients with documented clinical ASCVD (*n*** **=** **40)**	
Age 45–70 years	Major cardiovascular event within 30 days*
History of myocardial infarction	EF < 40% and/or heart failure NYHA ≥ III
Coronary revascularization	Glomerular filtration rate (eGFR) < 60 ml/min/1.73 m^2^
Arterial revascularization procedures	Active infection
History of Stroke	Antibiotics or probiotics use within 30 days
PAD	Active liver disease
	Malignancy
	Inflammatory diseases, e.g., intestinal bowel disease
	Unsigned informed consent
**Patients with risk factors (RFs) for atherosclerosis (*n*** **=** **40)**	
Age 45–70 years	Documented clinical ASCVD
At least one major vascular RF:	EF < 40% and/or heart failure NYHA ≥III
● Arterial hypertension	eGFR < 60 ml/min/1.73 m^2^
● Diabetes mellitus	Active infection
● Raised lipid levels or treatment for dyslipidemia	Antibiotics or probiotics use within 30 days
● Smoking	Active liver disease
● Family history of premature CVD	Malignancy
	Inflammatory diseases, e.g., intestinal bowel disease
	Unsigned informed consent
**Healthy controls (*n*** **=** **15)**	
Age 45–70 years	Unsigned informed consent
No established diseases	
No major vascular RFs from those previously listed	

**Abbreviations: **ASCVD, atherosclerotic cardiovascular disease; CVD, cardiovascular disease; EF, ejection fraction; NYHA, The New York Heart Association Classification for Heart failure; PAD, peripheral artery disease.

All patients provided informed consent prior to examinations, and the study was approved by the local ethics committee, protocol approval number 01312/2022. Participants received no compensation for their participation in this study.

### Data collection

Information relating to medical history, concomitant conditions, and therapy was gathered. Detailed information was obtained on RF profiles and duration – arterial hypertension, diabetes mellitus, smoking status, dyslipidemia, and premature history of CVD, as well as accompanying treatments. Determining the presence/absence of a major vascular RF (e.g., arterial hypertension, dyslipidemia, family CVD history and diabetes mellitus) was performed according to current European Guidelines [1].

In patients with a ASCVD history, we collected data related to the number and timing of events and revascularization procedures.

All patients underwent a standard physical examination and electrocardiogram (ECG). Also, in a quiet room after a 5 min rest in a supine position, three blood pressure (BP) measurements were performed 1–2 min apart and a final BP was recorded as the average of the last two measurements. Body mass index (BMI) was calculated as kg/m^2^, where kg indicated the patient’s weight and m^2^ indicated their height in meters squared.

### Laboratory analyses

Patients were fasted for laboratory analyses. We examined the following parameters; creatinine levels, lipid profiles, glycated hemoglobin (HbA1c), and glucose levels. The glomerular filtration rate (eGFR) was estimated using chronic kidney disease epidemiology collaboration formula and was represented in ml/min/1.73 m^2^. Laboratory assessments, including those for TMAO levels and imaging procedures, were performed on the same day.

### Imaging procedures

Arterial stiffness was measured by Carotid-Femoral Pulse Wave Velocity (C-F PWV) using applanation tonometry and a validated transfer function (SphygmoCor, Atcor Medical). The procedure was performed in a quiet room at 22°C with the patient in a supine position. A BP measurement was taken just before the procedure. A sequential record of pulse waves of the carotid and femoral arteries was performed and synchronized with an ECG. Then, the time difference between the arrival of the pulse wave in the carotid and femoral arteries was determined. The distance travelled by the two waves was measured as the difference in distance between the sternum and the point of record on carotid and femoral arteries on the body surface. PWV was automatically calculated in m/s.

**Figure 1:**
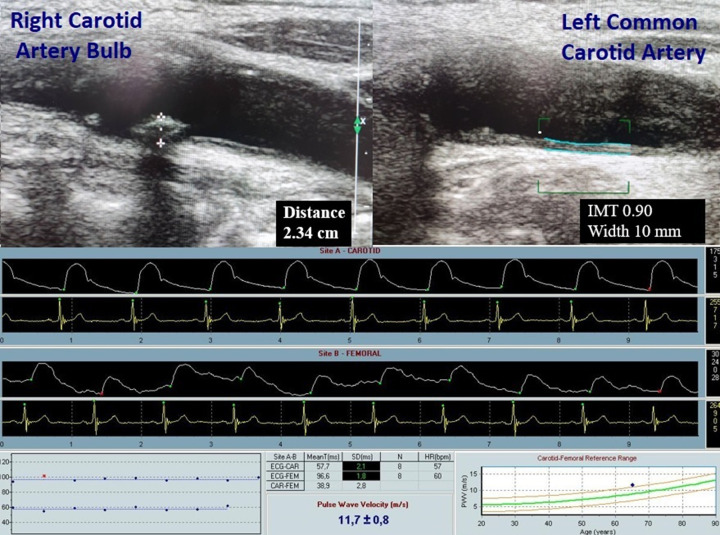
Measuring vascular alterations Measuring vascular structure and function parameters in a 65-year-old patient with a history of myocardial infarction, arterial hypertension, and dyslipidemia. Three plaques (one in the right carotid bulb, one in the right internal carotid artery, and one in the left internal carotid artery) were detected. Maximum plaque height was 2.34 cm and the plaque score was 3. The average intima media thickness (IMT) value between left and right common carotid arteries (CCAs) was 0.89. Pulse wave velocity was estimated as 11.7 m/s. The TMAO value was 317 µg/l.

B-mode ultrasonography was used to evaluate the presence and grade of CAPs (Figure 1) and for carotid intima media thickness (CIMT) [6,7].

Carotid examinations included the visualization of common, internal, and external carotid arteries and carotid bifurcation. A linear transducer at frequencies >7 MHz was used. Arterial wall segments were assessed in a transversal and longitudinal view, perpendicular to the ultrasound beam, with a depth of focus of 30–40 mm and a frame rate of 25 Hz. CIMT was measured on the far wall of the common carotid artery (CCA) at 5 mm below its end with automated analysis of a 10 mm segment in diastole and synchronized with the ECG. Mean values from the left and right CCA were used in analyses.

### Plaque measurements

A carotid artery plaque was defined as a focal thickening that encroached into the lumen by 0.5 mm, or by 50% of the surrounding IMT, or where CIMT ≥ 1.5 mm [7]. A grading system, based on American Society of Echocardiography (ASE) recommendations for CAP assessments by ultrasound, was used to determine plaque grades from 0 to III (Table 2).

**Table 2:** Grading carotid artery plaques (CAPs)

Grade	Plaque type	Plaque thickness
0	No plaque	CIMT < 1.5 mm
I	Protuberant thickening of vessel wall	<1.5 mm
II	Protruberant or diffuse; CIMT ≥ 1.5 mm	1.5–2.4 mm
III	Protruberant or diffuse (CIMT ≥ 2.5 mm)	≥ 2.5 mm

**Abbreviations: **CIMT, carotid intima-media thickness. Plaque grading was performed based on recommendations for assessing CAPs by ultrasound to characterize atherosclerosis and evaluate cardiovascular risk [7].

Plaque scores were assessed as the total number of sites containing plaques along the distal 1 cm of the CCA, carotid bifurcation, and the proximal 1 cm of the internal carotid artery. Plaque scores ranged from 0 to 6, and were estimated by adding the number of sites where plaques were detected, dividing by the total number of sites for which an ultrasound image was available, and then multiplying by 6.

Plaque height was measured using electrocardiographic gating from an adventitial-medial layer of the plaque origin into the lumen at rights angles to the wall. Single, largest plaque height was reported for analyses.

### TMAO measurements

Patients were fasted on the day of analysis. To reduce the influence of dietary factors on TMAO levels, patients were asked not to consume red meat, milk products, fish, and energy drinks within 2 days prior to examinations.

Using appropriate standards, high-performance liquid chromatography with triple quadrupole tandem mass spectrometry (HPLC-MS/MS) was developed and validated to determine ТМАО levels in human plasma at the Central Laboratory of Therapeutic Drug Management & Clinical Pharmacology at Alexander University Hospital, Faculty of Medicine, Medical University of Sofia, Bulgaria (Supplementary File S1) [8–10]. TMAO levels were determined in external standardization mode after the protein precipitation of plasma using acetonitrile. Chromatographic separation was performed on a RPC8 analytical column with isocratic elution, using a mobile phase consisting of methanol and ammonium fluoride buffer. Detection was performed using positive electrospray ionization and SRM-MS/MS monitoring. Raw data from MS chromatograms were collected and processed using specialized software ‘Xcalibur 1.4’. A weighted (1/X) linear regression was selected to determine TMAO concentrations, which were calculated in external standard mode using TMAO peak areas, with values expressed as µg/l.

### Statistical analysis

Statistical analyses were performed using SPSS23 for Windows software. Kolmogorov-Smirnov tests were used to assess variable distributions. Continuous variables with a normal distribution were presented as the mean ± standard deviation. For other variables, parameters were presented as the median and interquartile range (IQR). TMAO levels were with skewed distribution, and results were presented as the median and interquartile range (IQR) in brackets, where IQR is the difference between upper and lower quartile. Independent sample *t*- and Mann–Whitney two-sample tests were used to compare normally and abnormally distributed continuous variables, respectively. Categorical variables were presented as percentages and counts and analyzed using χ^2^ or Fisher’s exact tests. Kruskal–Wallis tests were used to compare skewed continuous variables in patient groups. Mann–Whitney tests were used for post-hoc analyses with Kruskal–Wallis tests. To examine differences between normally distributed continuous variables in patient groups, one-way analysis of variance tests were used and Bonferroni tests were applied to *post hoc* tests. Relationships between continuous normally distributed variables were determined using Pearson’s and Spearman’s correlation coefficients according to distribution.

The area under the receiver operating characteristic (ROC) curve was measured to estimate the ability of TMAO to predict the presence of CAPs.

A multinomial linear logistic regression analysis using the Enter method was performed to identify independent predictors of PWV, plaque height, and plaque score. Binary logistic regression analyses were used to identify TMAO as an independent predictor of high-grade plaques (grade III) from others. A two-tailed *P-*value < 0.05 was considered statistically significant.

## Results

### Patient demographics and clinical characteristics

The study population included 95 patients. The mean age was 58.5 ±7.4 years and 60 of patients (63.1%) were males. Fifty-one of patients (53.6%) were with obese, defined as BMI > 30 mg/kg m ^2^. We divided patients into three groups according to CVD RFs (Table 1). Then, we assessed relationships between TMAO and RF profiles and TMAO and vascular alterations (PWV, CIMT, and CAP).

### TMAO levels across CVD risk groups

We identified no statistically significant age difference between groups. The ratio of patients with male gender was higher in the study population, also not statistically significance between the groups according to the gender was observed. The main patient characteristics and RF profiles are shown (Table 3).

**Table 3:** Main patient characteristics and risk factor (RF) profiles

Parameter	Patients with ASCVD, *n*=40	Patients with RF, *n*=40	Controls, *n*=15	*P*
Age (years)	60.7 ± 8.1	57.1 ± 6.8	56.6 ± 5.9	ns
Gender % (males)	75% (30)	57.5% (23)	46.7% (7)	ns
SBP (mm/Hg)	138.2 ± 17.1*	130.7 ± 13.5*	115.3 ± 7.2	0.003
DBP	81.9 ± 8.6*	82.1 ± 9.1*	73.4 ± 5.5	0.006
Weight (kg)	91.3 ±14.9	90.6 ± 19.9	79.0 ±20.6	0.08
BMI (kg/m^2^)	31.2 ± 4.6	30.8 ± 5.7	27.3 ± 5.6	ns
Arterial hypertension % (*n*)	97.5% (39)	77.5% (31)	-	0.017
Dyslipidemia % (*n*)	77.5% (31)	70% (28)	-	ns
Family history % (*n*)	10% (4)	17.5% (7)	-	ns
DM % (*n*)	42.5% (17)	30% (12)	-	ns
Smoking % (*n*)	35% (14)	27.5% (11)	-	ns
Total cholesterol (mmol/l)	4.8 ± 1.4	5.9 ± 1.5*	5.0 ± 0.3	0.003
LDL cholesterol (mmol/l)	2.6 ± 1.1	3.7 ± 1.2*	2.8 ± 0.2	0.01
Non-HDL cholesterol (mmol/l)	3.6 ± 1.4	4.6 ±1.5*	3.4 ± 0.3	0.001
Triglycerides (mmol/l)	1.81 ± 1.2*	1.59 ± 0.53	0.82 ± 0.4	0.01
HBA1C (%)	5.95 (1.25)*	5.45 (0.75) *	5.25 (0.4)	0.003
Statin therapy (%)	80%(32)	30% (12)	-	< 0.001
DM on insulin therapy	10% (4)	5% (2)	-	< 0.001

*P<0.05 vs. controls; *P*<0.05 vs. patients with ASCVD.

Results are presented as the mean ± standard deviation. ANOVA tests with post-hoc Bonferroni tests were used to determine differences between normally distributed continuous variables. For TMAO levels, results are presented as the median and interquartile ranges. Kruskal–Wallis tests with post-hoc Mann–Whitney tests were used to present differences between continuous variables with a skewed distribution; ASCVD, atherosclerotic cardiovascular disease; BMI, body mass index; DBP, diastolic blood pressure; DM, diabetes mellitus; RF, risk factor; SBP, systolic blood pressure.

**Figure 2:**
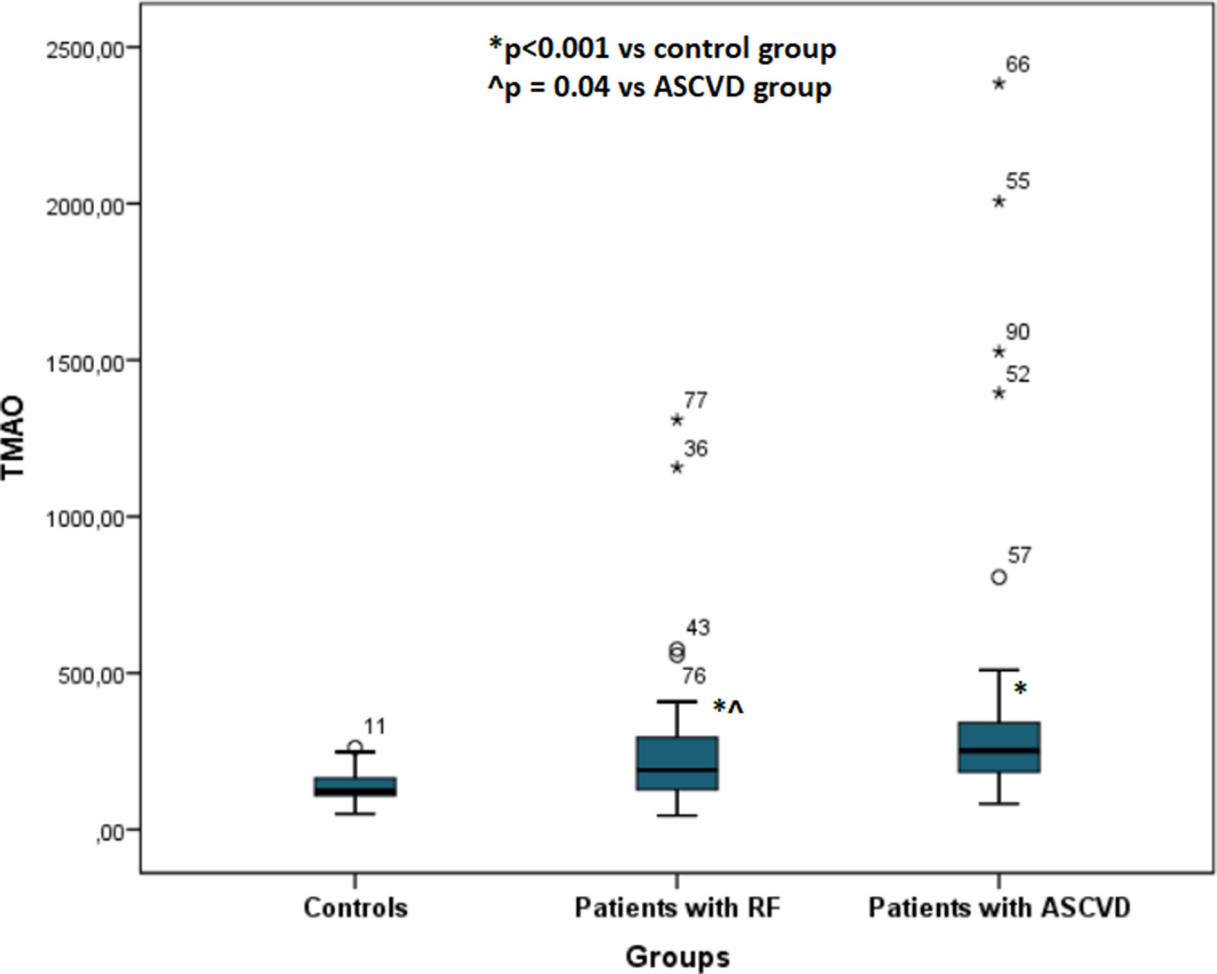
TMAO levels across different CVD risk groups *x*-axis: groups of patients: controls; patients with risk factors; patients with ASCVD. *y*-axis: TMAO levels in µg/l; ASCVD, atherosclerotic cardiovascular disease; RF, risk factor; TMAO, trimethylamine N-oxide.

TMAO levels were significantly increased in patients with ASCVD (251.5 [164.5] µg/l) when compared with patients with RFs alone (194.0 [174] µg/l, *P*=0.04) and controls 122.0 [77] µg/l, *P*<0.001) (Figure 2).

### TMAO levels and RFs

According to RF profiles in patients, TMAO levels were not significantly different in relation to arterial hypertension (*P*=0.87), dyslipidemia (*P*=0.39), smoking (*P*=0.55), and a family history of premature CVD (*P*=0.81). In patients with diabetes mellitus, TMAO levels were significantly increased when compared with patients without the condition (285 [129] vs. 210 [164], *P*=0.0032, vs. controls 122 [77], *P*<0.05).

### Correlation analysis between TMAO levels and patient clinical characteristics

In our study, TMAO levels were not significantly correlated with age (*P*=0.31) and associations between TMAO and BMI were not observed (*P*=0.22). There were no statistically significant differences in TMAO levels related to gender (208 [169] µg/l in males, vs. 214 [182] µg/l in females, *P*=0.63). No significant correlations were identified between low-density lipoprotein (LDL) (*P*=0.82), non-high-density lipoprotein (HDL) (*P*=0.52) levels, and TMAO. A mild, statistically significant association was identified between TMAO and triglycerides (*r* = 0.24, *P*=0.033) and HbA1C levels (*r* = 0.031, *P*=0.04).

### Vascular alterations in patients

Arterial stiffness (PWV), CIMT, CAP, plaque score, and plaque height values are shown (Table 4).

**Table 4:** Vascular structure and function parameters in patients

Parameter	Patients with ASCVD, *n*=40	Patients with RF, *n*=40	Controls, *n*=15	*P*
PWV, m/s	9.37 ± 2.00*	8.88 ± 2.02*	7.31 ± 0.7	0.00
CIMT, mm	0.75 ± 0.14*	0.70 ± 0.18	0.6 ± 0.1	0.01
Plaque score (0-6)	2.79 ± 1.96	0.84 ± 1.42	-	<0.001
Plaque height, mm	2.7 ± 1.58	2.03 ± 0.73	-	0.002
Presence of CAPs % (*n*)	85% (34)	42.5% (17)	-	<0.001

*P<0.05 vs. controls; *P*<0.05 vs. ASCVD group. ASCVD, atherosclerotic cardiovascular disease; CAP, carotid artery plaques; PWV, pulse wave velocity; RF, risk factors. ANOVA tests with *post hoc* Bonferroni tests were used to determine differences between continuous variables.

PWV levels were significantly increased in patients with ASCVD (9.37 ± 2.0 m/s) and in patients with RFs (8.88 ± 2.02 m/s) when compared with controls (7.31 ± 0.7 m/s), *P*=0.000. Patients with ASCVD had increased plaque score (*P*<0.001) and plaque height levels (*P*=0.002) when compared with the RF group. We observed that 34 patients (85%) in the ASCVD group and 17 (42.5%) in the RF group had CAPs, *P*<0.001.

### Correlations between TMAO, PWV, and CIMT values in patients

**Figure 3:**
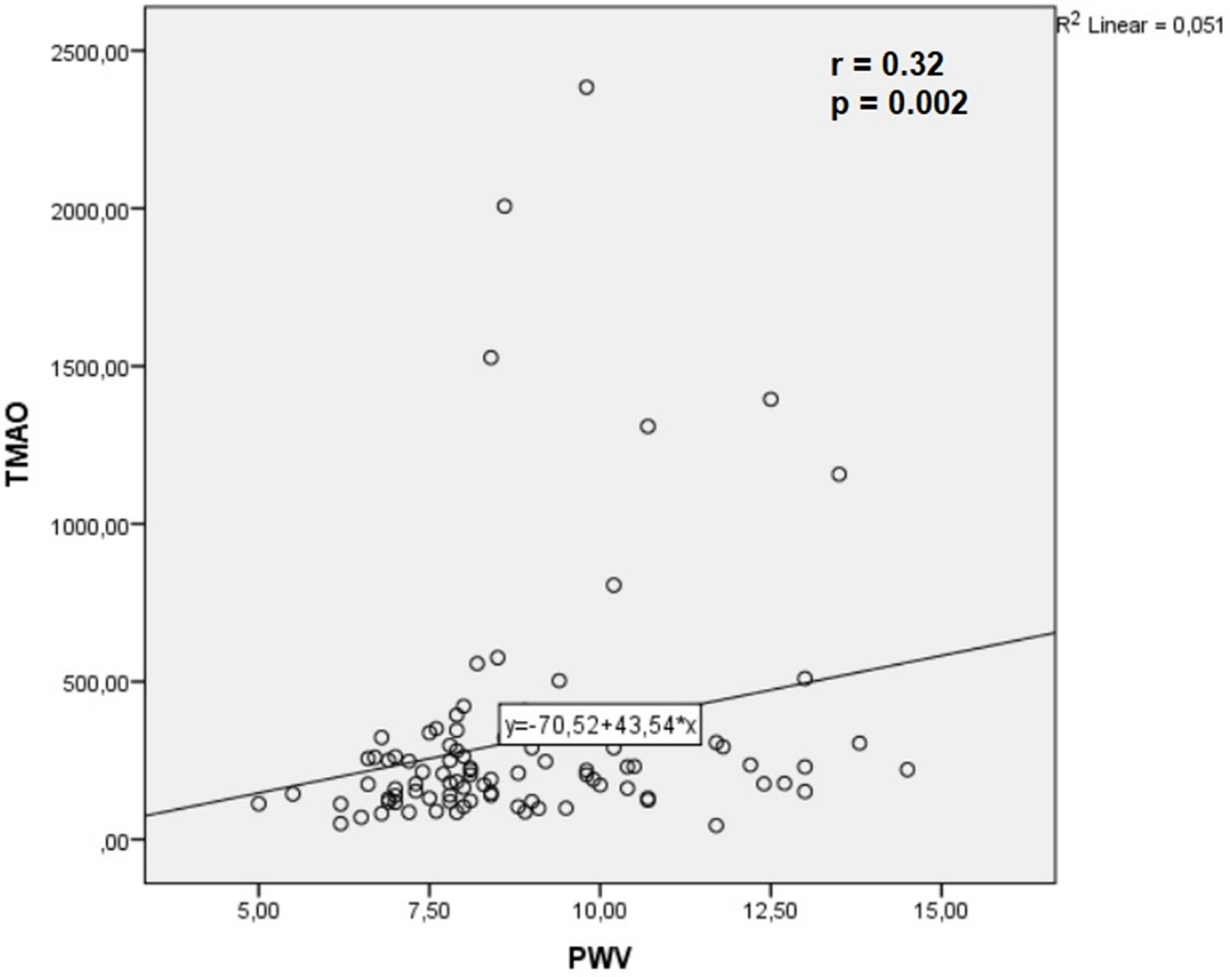
Correlation between TMAO and pulse wave velocity (PWV) *x*-axis: Pulse wave velocity in m/s. *y*-axis: TMAO levels in µg/l; PWV, pulse wave velocity; TMAO, trimethylamine N-oxide.

We observed a mild but statistically significant correlation between TMAO levels and PWV in all patients (*r* = 0.32, *P*=0.002) (Figure 3). A correlation between TMAO and CIMT was not observed (*P*=0.95).

### TMAO and CAPs

**Figure 4:**
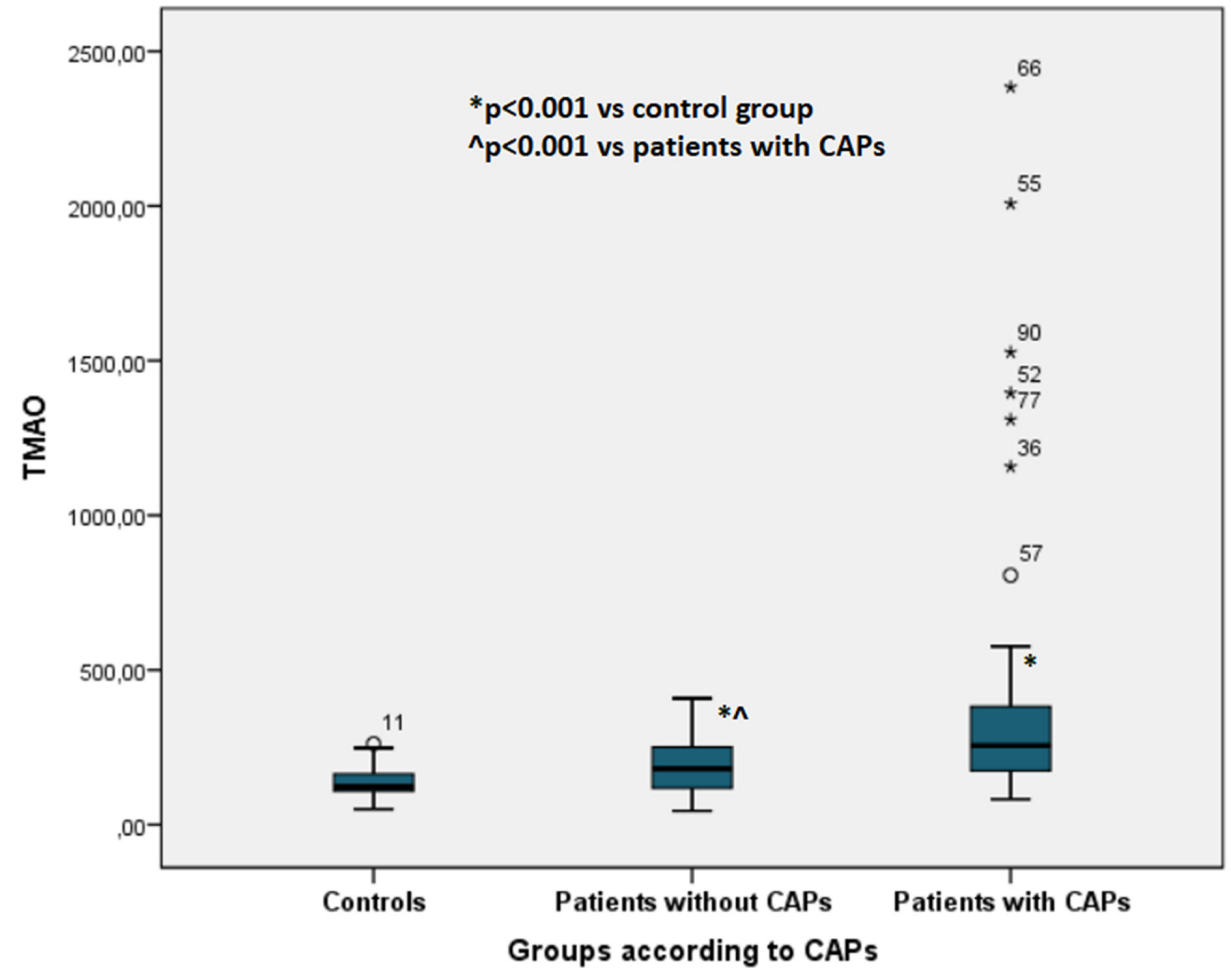
TMAO levels according to the presence/absence of carotid artery plaques (CAPs) *x*-axis: groups of patients according to CAPs: controls; patient without CAPs; patients with CAPs. *y*-axis: TMAO levels in µg/l; CAP, carotid artery plaque; TMAO, trimethylamine N-oxide.

Across the study population (*n*=95), 51 patients presented with CAPs. TMAO levels were increased in patients with CAPs when compared with those without (*n*=29), regardless of clinical ASCVD history (256 [213] µg/l vs. 181 [135] µg/l, *P*<0.001 vs. 122 [77] µg/l in controls, *P*<0.001) (Figure 4).

**Figure 5:**
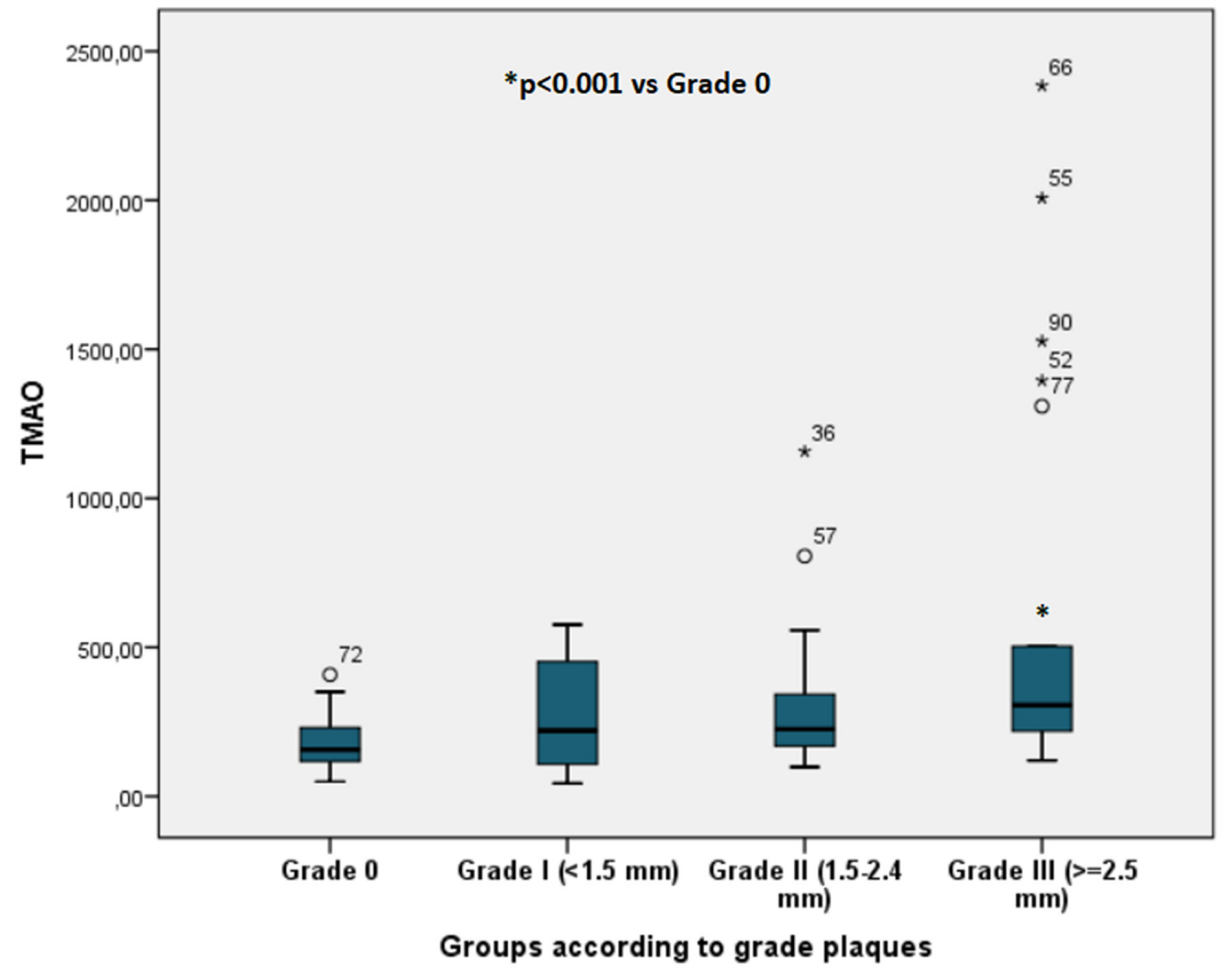
TMAO levels according to plaque grade *x*-axis: Groups of patients according to plaque grade: Patients without CAPs (Grade 0); patients with CAPs grade I (<1.5 mm); patients with CAPs grade II (<1.5–2.4 mm); patients with CAPs grade III (>2.5 mm). *y*-axis: TMAO levels in µg/l; CAP, carotid artery plaque; TMAO, trimethylamine N-oxide.

TMAO level differences according to plaque grade (0–III) are shown (Figure 5).

#### TMAO levels, plaque scores, and plaque height

A moderate but statistically significant correlation was observed between TMAO levels and plaque scores (*r* = 0.434, *P*=0.000) and plaque height (*r* = 0.479, *P*=0.000). Plaque height was also linked to other factors; age (*r* = 0.45, *P*=0.000), SBP (*r* = 0.46, *P*=0.000), and HbA1C (*r* = 0.21, *P*=0.044). A similar significant correlation for plaque scores was observed with age (*r* = 0.40, *P*=0.000), SBP (*r* = 0.43, *P*=0.000), and HbA1C (*r* = 0.29, *P*=0.007).

After we adjusted for RFs in multinominal linear regression analyses, TMAO values as independent predictors for plaque height and plaque scores were ᵦ 0.200, confidence interval (CI) 95%: 0.000025–0.001, *P*=0.04 for plaque height, and ᵦ 0.210, CI 95%: 0.00012–0.002, *P*=0.02 for plaque scores.

**Figure 6:**
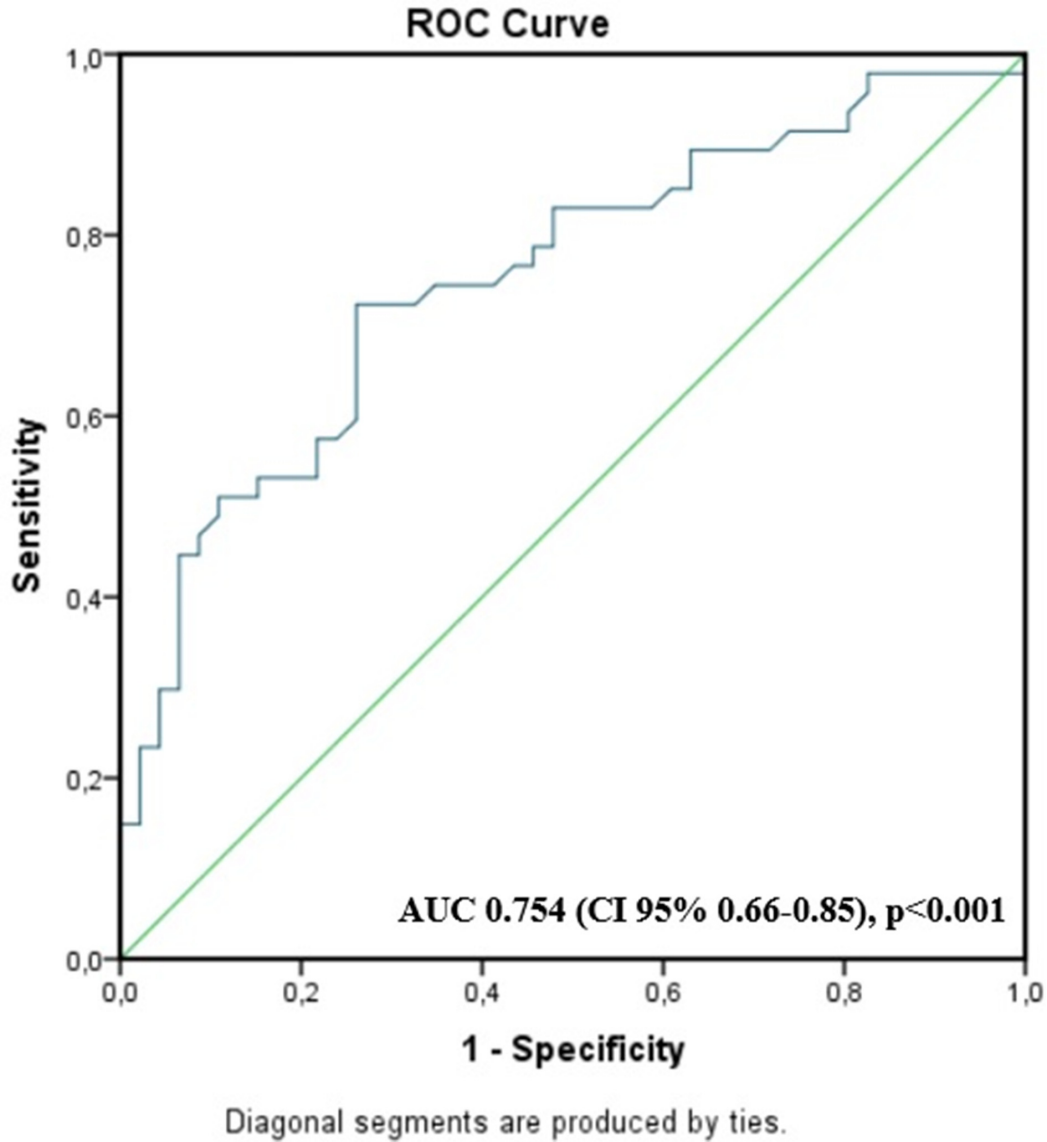
Receiver operating characteristic analysis showing that TMAO is an independent predictor of carotid artery plaques (CAPs) in patient population Specificity (*x*-axis) and sensitivity (*y*-axis) of TMAO (blue line) as an independent predictor of carotid artery plaques in examined patients; CAP, carotid artery plaque; TMAO, trimethylamine N-oxide.

Our ROC analyses showed that TMAO levels > 212 µg/l, with an area under the ROC curve = 0.754, *P*<0.001, identified patients with CAPs, with sensitivity = 70.2% and specificity = 73.9% (Figure 6).

### Regression analysis of TMAO as an independent predictor of PWV

PWV values were significantly correlated with age (*r* = 0.46, *P*=0.000), SBP (*r* = 0.48, *P*=0.000), TMAO (*r* = 0.47, *P*=0.003), and HbA1C (*r* = 0.33, *P*=0.002).

In multiple regression analyses, when we included age, SBP, TMAO, and HbA1C values, only age (β = 0.35; *P*=0.000) and SBP (β = 0.32; *P*=0.002) were independent predictors of PWV levels F (4.80) = 11.1; *R*^2^ = 0.36, *P*<0.01. TMAO values were not statistically significant (*P*=0.17).

### TMAO as an independent predictor of CAPs

**Figure 7:**
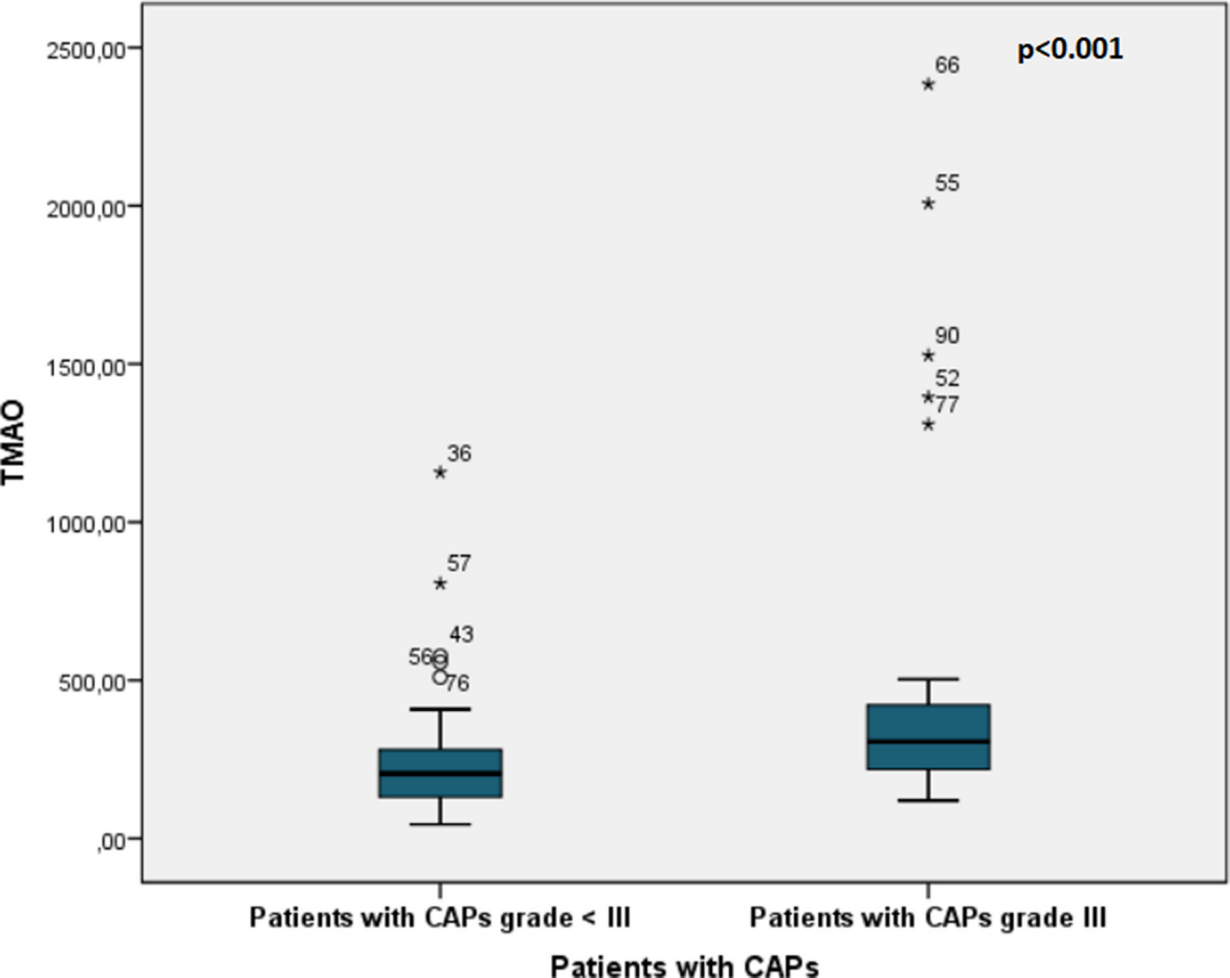
TMAO levels in patients with grade III carotid artery plaque (CAPs) (*n*=27) vs. patients with < III CAPs (*n*=68) *x*-axis: groups of patients: patients with CAPs <III; patients with CAPs grade III. *y*-axis: TMAO levels in µg/l; CAP, carotid artery plaque; TMAO, trimethylamine N-oxide.

TMAO levels were independent predictors for grade III CAPs when compared to other factors in binary logistic regression analyses (OR = 1.002, CI 95%: 1.000047–1.003, *P*=0.044) (Figure 7). This model included age, SBP, HbA1C, LDL cholesterol, and TMAO values. Age, HbA1C, and TMAO remained independent predictors for grade III CAPs.

## Discussion

### TMAO levels across different CVD risk groups and associations with RF profiles

As a phospholipid metabolite, TMAO is generated by the gut microbiota via the digestion of foods containing carnitine and phosphatidylcholine, and is a molecule that may be directly involved in CVD development and progression [4].

First described in 2011 by Wang et al., TMAO was established as an independent predictor of major adverse CVD events, which tended to correlate with the degree of coronary atherosclerosis [11]. To date, TMAO has been predominantly studied in very-high risk patients. However, a recent trial reported that higher TMAO levels, in 6785 patients free of clinical CVD at baseline, corresponded to increased risks of all-cause mortality and kidney failure [12]. In this trial, patients with increased BMI and SBP and slightly increased triglyceride and fasting glucose levels at baseline tended to have higher TMAO levels, suggesting early metabolite interactions with a number of CVD RFs [13].

We observed a significant increase in TMAO levels in the RF group when compared to controls. TMAO levels were also highest in patients with sustained ASCVD. Among RF parameter profiles, we detected positive correlations between TMAO levels, HbA1C levels, and a history of diabetes mellitus. Several mechanisms potentially interconnect the gut microbiome, inflammation, and insulin resistance. Most encompass changes in microbiota composition, with a reduced abundance of butyrate-producing bacteria, decreased gut permeability, and enhanced endotoxin release into the circulation, which promote an inflammatory state. TMAO can also induce hyperglycemia by binding to the protein-RNA like endoplasmic reticulum stress kinase to impair glucose tolerance [3,14,15]

We identified a significant positive correlation for TMAO with triglyceride levels, consistent with other studies [16,17]. It is important to note that TMAO levels are influenced by dietary habits, which may explain this association [18]. The relationship between TMAO levels and insulin resistance may also have important roles in this association [3,14,15].

We did not identify a significant association between TMAO levels and LDL-cholesterol levels. It is important to note that all patients with RFs received corresponding anti-cholesterol therapy, including statins. In our study, we did not evaluate the impact of therapy on TMAO levels and any associations with RFs and vascular abnormalities. Data from another trial demonstrated that statin therapy appeared to influence associations between TMAO and LDL-cholesterol levels [16].

Additionally, we did not identify a significant correlation between TMAO levels and age; however, our age limits (45–70 years) may have affected this association. In view of the fact that the study population was predominantly obese, we observed no significant correlations between TMAO and BMI.

### TMAO and vascular structure and function parameters

#### TMAO and arterial stiffness

Several mechanisms, including endothelial cell oxidative stress and inflammation and activated vascular smooth muscle cell calcification, are purportedly related to TMAO [5]. One study demonstrated that decreased gut microbiome diversity was associated with increased arterial stiffness in 617 middle-aged women, and that microbiome factors explained 8.3% of PWV variance [19]. We also identified a moderately significant TMAO association with PWV; however, this association was abolished after adjusting for other RFs.

In a previous study, a positive association between TMAO and CIMT was identified [20], but we observed no such relationship. It is important to state that CIMT is not solely a marker of atherosclerosis, it also reflects changes in the media of the artery and is better characterized as a combined parameter encompassing vascular aging, the effects of systolic hypertension, and atherosclerosis. Also, a previous meta-analysis showed that the diagnostic accuracy of CIMT as a predictor of CVD events was limited [21].

#### TMAO and CAPs

Several studies have reported links between TMAO levels and the degree of coronary artery disease [4,11,22]. Studies have also reported TMAO associations with imaging evidence of atherosclerosis, especially CAPs, but data are somewhat inconsistent [23,24].

We observed in patients with CAPs that TMAO levels were significantly increased, independent of ASCVD history. Furthermore, TMAO levels were a significant and independent predictor of CAPs in our ROC analyses.

We also observed that TMAO levels were positively correlated with a degree of carotid atherosclerosis, via associations with parameters such as a plaque height and plague scores, and remained an independent predictor of high-grade plaques. These observations suggested that TMAO levels could be indicators of atherosclerotic processes per se, and were potentially associated with a higher degree of atherosclerosis. Indeed, to date, the main TMAO mechanisms which contribute to CVD risk are predominantly related to LDL modification, foam cell formation, platelet reactivity, and inflammation [11,25]. Recently, TMAO was shown to have direct effects on platelets, through augmented stimulus dependent (thrombin, adenosin diphosphate and collagen) calcium release from intracellular stores and thereby enhancing platelet reactivity and thrombotic risk [26]. Moreover, some studies have reported that TMAO was related to plaque instability and rupture [27].

Many trials predominantly conducted in very high-risk patients have reported correlations between TMAO and CAD severity, as assessed by invasive coronary angiography [4,11,22,28]. However, results regarding associations between TMAO and modern non-invasive imaging methods, to assess cardiovascular risk and coronary atherosclerosis (coronary calcium score and coronary computed tomography angiography [CCTA]), are controversial [29,30]. Lower-risk populations in these studies may be one explanation for these results. The possibility to assess not only degree but also different features and characteristics of coronary artery plaques by CCTA sets the stage for future studies, aimed to investigate the association of TMAO with different types of plaques and in different patient populations.

These findings provide a platform to examine and identify the effects of TMAO on vascular pathobiology and CVD risk. Our study has demonstrated that patients with atherosclerotic events and enhanced atherosclerotic burden have increased TMAO levels. However, future studies must assess the role and significance of TMAO as an early detector of CVD in younger populations. Whether diminished TMAO levels reduce CVD risk remains a key question for future research.

### Study strengths

The prognostic effects of TMAO have been observed in subsets of patient with heart failure, including ischemic and nonischemic etiologies, chronic kidney failure, acute coronary syndromes, and chronic coronary artery diseases, all independent of traditional RFs [31–33]. However, the pathomorphology substratum and pathophysiology effects of TMAO on vascular function, if they exist, remain unknown.

Our study strengths included the comprehensive investigation of different vascular function indices in our patient cohort. On one hand arterial stiffness, a marker of vascular aging, predominantly associated with changes in the media of the arteries. On the other, the presence and grade of carotid artery plaques, which is imaging evidence of atherosclerosis.

Also, our study population comprised patients with preserved renal function, the selection of which was based on previous evidence that TMAO is excreted by the kidneys [34], and as a consequence, eGFR was eliminated as a significant confounder in our analyses. Age limits in our study reduced the significance of this factor in investigating vascular function parameters.

### Study limitations

Our study had several limitations. First, it was a single-center study and the patient population was small. Our cross-sectional analysis did not allow us to conclude the causal effects of TMAO. All patients received corresponding therapy, the effects of which were not evaluated in our analysis. Also, TMAO levels are affected by diet, but dietary habits were not considered. For atherosclerosis assessments, we used only carotid ultrasound in analyses; however, atherosclerosis processes can start on other artery branches, which were not evaluated. Also, one parameter that we used to assess vascular alterations was CIMT, which is now recognized with low reproducibility and sensitivity for CV risk predictions.

## Conclusion

TMAO levels are increased with expanded CVD risk. Among different types of vascular damage, TMAO levels are associated with atherosclerotic changes, especially with a higher degree of atherosclerosis.

## Supplementary Material

Supplementary File S1

## Data Availability

Data available within the article and its supplementary files. Additional data (demographic characteristics and TMAO levels in all examined patients) is provided on figshare.com: Spasova, Natalia (2024). Dataset. figshare. Dataset. https://doi.org/10.6084/m9.figshare.25601166.v1

## Abbreviations

ASCVD: atherosclerotic cardiovascular disease
CAP: carotid artery plaque
CVD: cardiovascular disease
C-F PWV: Carotid-Femoral Pulse Wave Velocity
CIMT: carotid intima-media thickness
DBP: diastolic blood pressure
DM: diabetes mellitus
IMT: intima-media thickness
LC-MS/MS: liquid chromatography-tandem mass spectrometry
LDL: low-density lipoprotein
RF: risk factor
SBP: systolic blood pressure
TMAO: trimethylamine N-oxide
