# Supplementary material for: Association of the trimethylamine N-oxide with cardiovascular risk and vascular alterations in middle-aged patients with risk factors for cardiovascular diseases
Source: Biosci Rep. 2024 May 24;44(5):BSR20232090. doi: 10.1042/BSR20232090 (PMC12046062; doi:10.1042/BSR20232090)
Supplement: Supplementary File S1 [file BSR-2023-2090_supp.pdf]

CLINICAL LABORATORY & CLINICAL PHARMACOLOGY
UNIVERSITY HOSPITAL ALEXANDROVSKA, FACULTY OF MEDICINE
MEDICAL UNIVERSITY OF SOFIA
1431, "Sv. Georgi Sofyiski" Str. № 1, phone:+359 2 9230929
e-mail: mbal@alexandrovska-hospital.bg

ANALYRICAL REPORT

Objective	Description of the validation experiments and project results for the determination of concentrations of Trimethylamine N-oxide in human plasma
Analyte	Trimethylamine N-oxide (TMAO)
Matrix	Human plasma
Method	HPLC/MS/MS
Lower limit of quantification	5.025 µg/L

Director:

Dobrin A. Svinarov, MD, PhD, Dr.Med.Sc.:

Professor and Head, Clinical Laboratory & Clinical Pharmacology,
Alexander Univ. Hospital, Faculty of Medicine
Medical University of Sofia,
Sofia, Bulgaria.

TABLE OF CONTENTS

1 SUMMARY 4

2 ANALYTICAL PROCEDURES 6

2.1 PRINCIPLE 6
2.2 REFERENCE STANDARDS 6
2.3 MATERIALS 6
2.4 EQUIPMENT 6
2.5 SOLUTIONS 7
2.6 SAMPLE PREPARATION 10
2.7 LIQUID CHROMATOGRAPHY-TANDEM MASS SPECTROMETRY 11

List of Abbreviations

TMAO	Trimethylamine N-oxide
CE	Collision energy
CV%	Coefficient of Variation (relative standard deviation: standard deviation/mean x 100) – used to describe imprecision
d%	Coefficient of Deviation (experimental concentration – theoretical concentration / theoretical concentration) x 100 – used to describe inaccuracy
g	Gram (mass)
HPLC	High Performance Liquid Chromatography
L	Liter (volume)
LLOD	Lower Limit of Detection
LLOQ	Lower Limit of Quantification
mg, µg, ng	Milligram (10^{-3} gram), Microgram (10^{-6} gram), Nanogram (10^{-9} gram)
min	Minute (time)
mL, µL	Milliliter (10^{-3} liter), microliter (10^{-6} liter)
M / mM	Mole, molar / Millimole, millimolar
MS/MS	Tandem mass spectrometry
m/z	mass to charge ratio
QCs (L, M, H)	Quality Control samples (Low, Middle, High)
S/N	Signal to noise ratio
SRM	Selected Reaction Monitoring
°C	Degrees centigrade
%	Per cent (part of hundred)
Vol	Volunteer
S	Sample
ULOQ	Upper Limit of Quantification
Re	recovery

1431, “Sv. Georgi Sofyiski” Str. № 1, phone:+359 2 9230929
e-mail: mbal@alexandrovska-hospital.bg

1 Summary

A high-performance liquid chromatographic method with triple quadrupole tandem mass spectrometric detection (HPLC-MS/MS) was developed and validated for the determination of Trimethylamine N-oxide (TMAO) in human plasma by the Central Laboratory of Therapeutic Drug Management & Clinical Pharmacology at Alexander University Hospital, Faculty of Medicine, Medical University of Sofia, according to SOP: TDMCP/ALHOSP.010 “Validation of chromatographic procedures with conventional or tandem mass spectrometric detection”[5.3] and in compliance with the EMA/CHMP//ICH /172948/2019 Guideline [5.1] and FDA Guidance for Industry[5.2], cited in the References Section of this report. The method was applied for the analysis of patient samples according to a project of the Cardiology Clinic, UMHAT “Tsaritsa Yoanna - ISUL”, MU-Sofia

One of the considerable challenges of TMAO quantitation is the high concentration of endogenous TMAO in typical biological matrices such as plasma or serum [5.5]. In this case, to avoid endogenous influence over the calibration procedure, a surrogate matrix is more suitable for the sensitive quantitation of low-concentration samples [5.6]. Therefore, we prepared a calibration standards and quality control samples for validation purposes in a surrogate matrix, instead of human plasma.

Determination of TMAO was performed in external standardization mode after protein precipitation of plasma with Acetonitrile. Chromatographic separation was performed on a RPC8 analytical column with isocratic elution, utilizing mobile phase, consisting of methanol and ammonium fluoride buffer. Detection was carried out on a tandem mass spectrometer with positive electrospray ionization and SRM - MS/MS monitoring. Raw data of the mass chromatograms were collected and processed by specialized software “Xcalibur 1.4”. A weighted (1/X) linear regression was selected to determine the concentration of the analyte. Concentrations were calculated in external standard mode, utilizing the peak area of TMAO and were expressed as µg/L.

CLINICAL LABORATORY & CLINICAL PHARMACOLOGY
UNIVERSITY HOSPITAL ALEXANDROVSKA, FACULTY OF MEDICINE
MEDICAL UNIVERSITY OF SOFIA
1431, "Sv. Georgi Sofyiski" Str. № 1, phone:+359 2 9230929
e-mail: mbal@alexandrovska-hospital.bg

The developed method demonstrates acceptable performance as outlined by TDMCP SOPs and is suitable for the determination of TMAO in human plasma over the range from 5,025 to 4020 µg /L.

A summary of the validation results is presented here as follows:

Calibration Curve Range	5,025 to 4020 µg/L
Linearity	$R^2 \geq 0.9978$
Within-Run Accuracy	QC d %: -3.2 to 6.5 %
Within -Run Precision	QC CV %: 1.57 to 6.1 %
Between-Run Accuracy	QC d %: 0.69 to 5.46 %
Between-Run Precision	QC CV %: 2.8 to 3.68 %
Recovery of TMAO	QC means: 95 to 105 %
Lower limit of Quantification (LLOQ)	5.025 µg/L with a S/N ratio of over 5/1
	accuracy and precision within ± 20%
Freeze–Thaw Stability Accuracy	d %: -1.0% to 3.0% at -20°C for 3 x 24 h

2 Analytical procedures

2.1 Principle

Determination of TMAO in human plasma was performed in external standardization mode, after protein precipitation with acetonitrile. Chromatographic separation was carried out on a RPC8 analytical column with isocratic elution, utilizing mobile phase consisting of methanol and ammonium fluoride buffer. Detection was carried out on a tandem mass spectrometer with positive electrospray ionization and SRM MS/MS monitoring of the protonated molecular ion of TMAO, decomposing under controlled conditions to the most dominant respective fragments. Raw data of the mass chromatograms were collected and processed by specialized software “Xcalibur 1.4”. Concentrations were calculated in external standard mode and expressed as µg/L.

2.2 Reference standards

Trimethylamine N-oxide (TMAO) , purity 98%, 317594-1G, Lot # SHBG6817V, Sigma-Aldrich.

2.3 Materials

Acetonitrile for HPLC, Super Gradient Reagent	VWR
Water for HPLC LC-MS grade	VWR
Methanol for LC-MS	PanReac
Ammonium Fluoride (NH_4F) 98% for LC-MS	Fluka
Bovine serum albumin (BSA),	Merck/Sigma, Lot 9048-46-8
Phosphate Buffered Saline (PBS), pH=7.4, 10g/L	Merck/Sigma, Lot SLCK1891
Колона Kinetex RPC8, 2.6µm p. size, 50mm x 2.1mm	Phenomenex, Part №008-4497-AN

2.4 Equipment

UltiMate 3000 Pump	Dionex
UltiMate 3000 Autosampler Dionex	Dionex
UltiMate 3000 Column Compartment	Dionex
TSQ Vantage	Thermo Scientific, USA

CLINICAL LABORATORY & CLINICAL PHARMACOLOGY
UNIVERSITY HOSPITAL ALEXANDROVSKA, FACULTY OF MEDICINE
MEDICAL UNIVERSITY OF SOFIA
1431, “Sv. Georgi Sofyiski” Str. № 1, phone:+359 2 9230929
e-mail: mbal@alexandrovska-hospital.bg

(triple quadrupole mass spectrometer)	
Computer DELL PRECISION T3500	Dell Computers, USA
Printer, Laser Jet Pro MFP M125a	Hewlett Packard, USA

2.5 Solutions

2.5.1 Preparation of Phosphate Buffered Saline (PBS) with concentration 10 g/L in deionized water - 0.5 g of **PBS** were weighted and transferred into a 50.0 mL class A volumetric flask. The flask is filed up to the mark with deionized water.

2.5.2 2.5.2 Preparation of surrogate matrix with concentration 80 g/L Bovine serum albumin (BSA) in PBS – 4.0 g of **BSA** were weighted and transferred into a 50.0 mL class A volumetric flask. The flask is filed up to the mark with **PBS** solution [2.5.1]

Mobile Phase- Methanol : 0.02 mM NH4F/10:90:

2.5.3 Preparation of 1M NH_4F buffer: 0.37037g of NH4F were weighted and dissolved in 10.0 mL of deionized water (class A volumetric flask).

2.5.4 Preparation of 0.02 mM NH4F buffer– 20 µL of 1M NH4F [2.5.3] were added to 100.0 mL of deionized water

2.5.5 Preparation of Mobile Phase- Methanol : 0.02 mM NH4F/10:90 – 10.0 ml of Methanol are mixed with 90.0 mL of 0.02mM NH4F

A. Preparation of Stock solutions of TMAO:

A1. Stock solution of TMAO (98%) with concentration 507 mg/L in methanol - 5mg (0.005g) of reference standard TMAO were weighted and dissolved in 10.0 mL of methanol (class A volumetric flask).

A2. Stock solution of TMAO (98%) with concentration 201 mg/L in methanol- 4mg (0.004g) of reference standard TMAO were weighted and dissolved in 20.0 mL of methanol (class A volumetric flask).

1431, "Sv. Georgi Sofyiski" Str. № 1, phone:+359 2 9230929
e-mail: mbal@alexandrovska-hospital.bg

B. Preparation of calibration standards (CC_01÷CC_06) of TMAO from stock solution [A2] – Table 1:

B1. Preparation of calibration solution CC_06 with concentration 4020 µg/L TMAO in a surrogate matrix - 100µL (0.1mL) from **Stock solution [A2]** were added to 5.00 mL of surrogate matrix [2.5.2] (class A volumetric flask).

B2. Preparation of calibration solution CC_05 with concentration 2010 µg/L TMAO in a surrogate matrix- 1.0 mL from solution **[B1]** are mixed with 1.0 mL of surrogate matrix [2.5.2].

B3. Preparation of calibration solution CC_04 with concentration 502.5 µg/L TMAO in a surrogate matrix- 0.5 mL from solution **[B2]** are mixed with 1.5 mL of surrogate matrix [2.5.2].

B4. Preparation of calibration solution CC_03 with concentration 100.5 µg/L TMAO in a surrogate matrix- 0.5 mL from solution **[B3]** are mixed with 2.0 mL of surrogate matrix [2.5.2].

B5. Preparation of calibration solution CC_02 with concentration 50.25 µg/L TMAO in a surrogate matrix- 1.0 mL from solution **[B4]** are mixed with 1.0 mL of surrogate matrix [2.5.2].

B6. Preparation of calibration solution CC_01 with concentration 5.025 µg/L TMAO in a surrogate matrix- 200µL (0.2mL) from solution **[B5]** are mixed with 1.8 mL of surrogate matrix [2.5.2].

C. The upper calibration range was set after the assessment of the analytical measurement interval (AMI) with 12 standards of TMAO (AMI_01÷AMI_12) encompassing concentrations between 1.0 ÷ 10 000 µg/L:

AMI1. Preparation of AMI _12 solution with concentration 10140 µg/L in a surrogate matrix- 100µL (0.1mL) from stock solution **[A1]** were dissolved in 5.00mL of surrogate matrix [2.5.2] (class A volumetric flask).

AMI 2. Preparation of AMI_11 solution with concentration 5070 µg/L in a surrogate matrix- 1.0 mL from solution **[AMI1]** are mixed with 1.0 mL of surrogate matrix [2.5.2].

AMI 3. Preparation of AMI _10 solution with concentration 2028 µg/L in a surrogate matrix- 1.0 mL from solution **[AMI 2]** are mixed with 1.5 mL of surrogate matrix [2.5.2].

AMI 4. Preparation of AMI _09 solution with concentration 1014 µg/L in a surrogate matrix- 1.0 mL from solution **[AMI 3]** are mixed with 1.0 mL of surrogate matrix [2.5.2].

AMI 5. Preparation of AMI _08 solution with concentration 507 µg/L in a surrogate matrix- 1.0 mL from solution **[AMI 4]** are mixed with 1.0 mL of surrogate matrix [2.5.2].

AMI 6. Preparation of AMI _07 solution with concentration 202.8 µg/L in a surrogate matrix- 1.0 mL from solution **[AMI 5]** are mixed with 1.5 mL of surrogate matrix [2.5.2].

AMI 7. Preparation of AMI _06 with concentration 101.4 µg/L in a surrogate matrix- 1.0 mL from solution **[AMI 6]** are mixed with 1.0 mL of surrogate matrix [2.5.2].

AMI 8. Preparation of AMI _05 solution with concentration 50.7 µg/L in a surrogate matrix- 1.0 mL from solution **[AMI 7]** are mixed with 1.0 mL of surrogate matrix [2.5.2].

AMI 9. Preparation of AMI _04 solution with concentration 20.28 µg/L in a surrogate matrix- 1.0 mL from solution **[AMI 8]** are mixed with 1.5 mL of surrogate matrix [2.5.2].

AMI 10. Preparation of AMI _03 solution with concentration 10.14 µg/L in a surrogate matrix- 1.0 mL from solution **[AMI 9]** are mixed with 1.0 mL of surrogate matrix [2.5.2].

AMI 11. Preparation of AMI _02 solution with concentration 5.07 µg/L in a surrogate matrix- 1.0 mL from solution **[AMI 10]** are mixed with 1.0 mL of surrogate matrix [2.5.2].

AMI 12. Preparation of AMI _01 solution with concentration 1.014 µg/L in a surrogate matrix- 0.5 mL from solution **[AMI 11]** are mixed with 2.0 mL of surrogate matrix [2.5.2].

D. Preparation of control samples QC_L, QC_M, QC_H and LLOQ (Table 2):

D1. Preparation of control sample QC_L with concentration 15.21 µg/L - 0.5 m from **[AMI 9]** solution are mixed with 0.5 mL from calibration solution **[AMI 10].**

D2. Preparation of control sample QC_M with concentration 1014 µg/L -1.0 mL from **[AMI3]** solution are mixed with 1.0 mL of surrogate matrix [2.5.2].

D3. Preparation of control sample QC_H with concentration 3549 µg/L -0.5 mL from **[AMI2]** solution are mixed with 0.5 mL from calibration solution **[AMI 3].**

D4. Preparation of control sample LLOQ with concentration 5.025 µg/L -200 µL (0.2mL) from calibration solution **[B5]** are mixed with 1.8 mL of surrogate matrix [2.5.2].

All samples for the validation procedures were divided into parts for daily use and were kept frozen below –20°C (exactly like patient plasma samples) until analysis.

Table 1. Preparation of Calibration Standards (CC_1÷ CC_6)

**Calibration standards**	Concentration [µg /L]	Type/Volume [mL] from respective solutions of TMAO	Volume [mL] of surrogate matrix	Total volume [mL]
CC_6	4020	**A2/0,1**	**5,0**	5,0
CC_5	2010	**B1/1,0**	**1,0**	2,0
CC_4	502,5	**B2/0,5**	**1,5**	2,0
CC_3	100,5	**B3/0,5**	**2,0**	2,5
CC_2	50,25	**B4/1,0**	**1,0**	2,0
CC_1	5,025	**B5/0,2**	**1,8**	2,0

Table 2. Preparation of QC Samples and LLOQ Sample

**Control materials**	Concentration [µg/ L]	Type/Volume [mL] from respective calibration solutions of TMAO	Volume [mL] of surrogate matrix	Total volume [mL]
LLOQ	5,025	**B5/0,2**	**1,8**	2,0
QC_L	15,21	**AMI9/0,5 + AMI10/0,5**		1,0
QC_M	1014	**AMI 3/1,0**	**1,0**	2,0
QC_H	3549	**AMI2/0,5 + AMI3/0,5**		1,0

2.6 Sample preparation

Fifty µL from each sample (CC, QC, patient derived) were transferred into a centrifugal tube of volume 1,5 mL; protein precipitation was performed by addition of 200 µL of acetonitrile, mixing and centrifugation; 20 µL from the supernatant were

transferred to second tube containing 200 µL of deionized water; sample was mixed again and 100 µL were transferred into a 250 µL volume insert for LC-MS/MS analysis. The injected amount was 10 µL.

2.7 Liquid chromatography-tandem mass spectrometry

Chromatography was performed on a Kinetex RPC8 column, 50mm x 2.1 mm, 2,6 µm particle size with mobile phase, consisting of 10% methanol and 90 % of 0.02 mM ammonium fluoride buffer. The chromatographic run time was 2.5 min with a retention time for the peak of TMAO of about 1.34 min. Detection was performed in electrospray positive ion mode. MS/MS monitoring of the column effluent at SRM mode was set to follow the predominant transitions: CE (collision energy) =18 at m/z 76. → 58. Raw data of the mass chromatograms were collected and processed by the specialized software "Xcalibur 1.4." All concentrations were calculated in external standard mode with use of the six-point calibration curve; area of the mass spectrometric peak of TMAO was used as a quantitative measure. Concentration units were µg/L. Representative mass spectrograms are presented on Figure 1 at the end of this report.

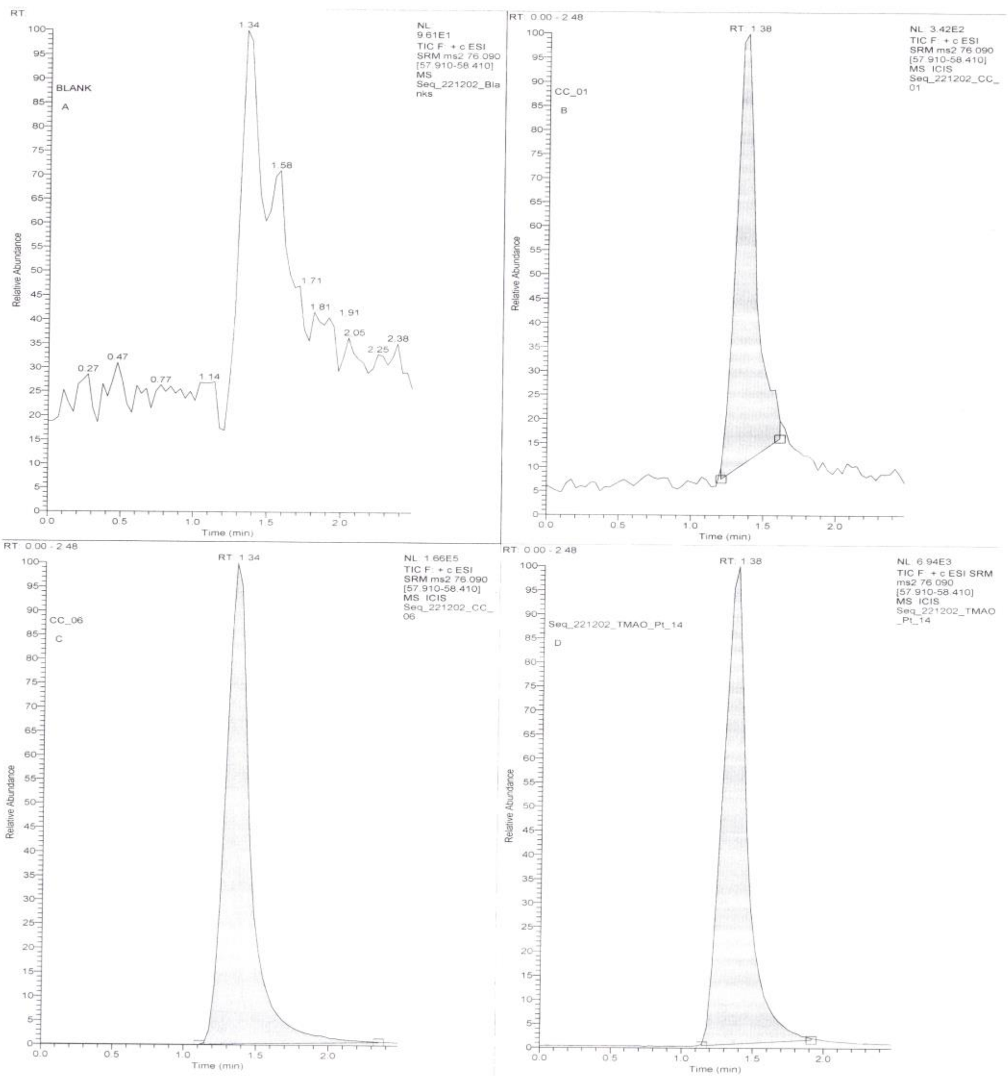

Figure 2. Mass spectrograms, obtained from TSQ Vantage, presenting the area of chromatographic peak of TMAO in a blank sample **(A)**, at the lowest (CC_01, **B**) and highest (CC_06, **C**) calibration points of the calibration range, as well as in patient sample №14 **(D)**, TMAO 162 µg/L.

2. References

5.1 EMA/CHMP/ICH/172948/2019: ICH guideline M10 on bioanalytical method validation and study sample analysis; 25 July 2022 .

5.2 FDA/ CDER /CBER: ICH M10 Bioanalytical method validation and study sample analysis; Guidance for Industry; November 2022 ICH

5.3 TDMCP/ALHOSP.SOP.010: "Validation of chromatographic procedures with conventional or tandem mass spectrometric detection"

5.4 Wang Z, Levison BS, Hazen JE, Donahue L, Li XM, Hazen SL. Measurement of trimethylamine-N-oxide by stable isotope dilution liquid chromatography tandem mass spectrometry. Anal Biochem 2014; 455:35-40.

5.5 Jones BR, Schultz GA, Eckstein JA, Ackermann BL. Surrogate matrix and surrogate analyte approaches for definitive quantitation of endogenous biomolecules. Bioanalysis 2012; 4:2343-2356.

Figure legends:

Fig. 1. Measuring vascular alterations

Measuring vascular structure and function parameters in a 65-year-old patient with a history of myocardial infarction, arterial hypertension, and dyslipidemia. Three plaques (one in the right carotid bulb, one in the right internal carotid artery, and one in the left internal carotid artery) were detected. Maximum plaque height was 2.34 cm and the plaque score was 3. The average intima media thickness (IMT) value between left and right common carotid arteries (CCAs) was 0.89. Pulse wave velocity was estimated as 11.7 m/s. The TMAO value was 317 μg/l.

Fig. 2. TMAO levels across different CVD risk groups

x-axis: groups of patients: controls; patients with risk factors; patients with ASCVD
y-axis: TMAO levels in μg/l.
ASCVD - atherosclerotic cardiovascular disease. **RF** – risk factors. **TMAO** – Trimethylamine N-oxide.

Fig. 3. Correlation between TMAO and pulse wave velocity (PWV)

x-axis: Pulse wave velocity in m/s
y-axis: TMAO levels in μg/l
TMAO - Trimethylamine N-oxide. **PWV** – pulse wave velocity

Fig. 4. TMAO levels according to the presence/absence of carotid artery plaques (CAPs)

x-axis: groups of patients according to CAPs: controls; patient without CAPs; patients with CAPs
y-axis: TMAO levels in μg/l
TMAO – Trimethylamine N-oxide. **CAPs -** carotid artery plaques

Fig. 5. TMAO levels according to plaque grade

x-axis: Groups of patients according to plaque grade: Patients without CAPs (Grade 0); patients with CAPs grade I (<1.5 mm); patients with CAPs grade II (<1.5 – 2.4 mm); patients with CAPs grade III (>2.5 mm)
y-axis: TMAO levels in μg/l
TMAO – Trimethylamine N-oxide. **CAPs -** carotid artery plaques

Fig. 6. Receiver Operating Characteristic analysis showing that TMAO is an independent predictor of carotid artery plaques (CAPs) in patient population

Specificity (x-axis) and sensitivity (y-axis) of TMAO (blue line) as an independent predictor of carotid artery plaques in examined patients.
TMAO – Trimethylamine N-oxide. **CAPs -** carotid artery plaques

Fig. 7. TMAO levels in patients with grade III carotid artery plaque (CAPs) (n = 27) vs. patients with < III CAPs (n = 68)

x-axis: groups of patients: patients with CAPs <III ; patients with CAPs grade III
y-axis: TMAO levels in µg/l
TMAO – Trimethylamine N-oxide. **CAPs -** carotid artery plaques
